# Evaluation of Aldose Reductase, Protein Glycation, and Antioxidant Inhibitory Activities of Bioactive Flavonoids in *Matricaria recutita* L. and Their Structure-Activity Relationship

**DOI:** 10.1155/2018/3276162

**Published:** 2018-04-10

**Authors:** Seung Hwan Hwang, Zhiqiang Wang, Yanymee N. Guillen Quispe, Soon Sung Lim, Jae Myung Yu

**Affiliations:** ^1^Department of Food Science and Nutrition, Hallym University, 1 Hallymdeahak-gil, Chuncheon, Republic of Korea; ^2^College of Public Health, Hebei University, Baoding 071002, China; ^3^Institute of Korean Nutrition, Hallym University, 1 Hallymdeahak-gil, Chuncheon, Republic of Korea; ^4^Institute of Natural Medicine, Hallym University, 1 Hallymdeahak-gil, Chuncheon, Republic of Korea; ^5^Hallym University Kangnam Sacred Heart Hospital, 1 Singil-ro, Yeoungdeungpo-gu, Seoul, Republic of Korea

## Abstract

The inhibitory activities of *Matricaria recutita* L. 70% methanol extract were evaluated by isolating and testing 10 of its compounds on rat lens aldose reductase (RLAR), advanced glycation end products (AGEs), and 2,2-diphenyl-1-picrylhydrazyl (DPPH) radical scavenging. Among these compounds, apigenin-7-*O*-*β*-D-glucoside, luteolin-7-*O*-*β*-D-glucoside, apigenin-7-*O*-*β*-D-glucuronide, luteolin-7-*O*-*β*-D-glucuronide, 3,5-*O*-di-caffeoylquinic acid, apigenin, and luteolin showed potent inhibition, and their IC_50_ values in RLAR were 4.25, 1.12, 1.16, 0.85, 0.72, 1.72, and 1.42 *μ*M, respectively. Furthermore, these compounds suppressed sorbitol accumulation in rat lens under high-glucose conditions, demonstrating their potential to prevent sorbitol accumulation ex vivo. Notably, luteolin-7-*O*-*β*-D-glucuronide and luteolin showed antioxidative as well as AGE-inhibitory activities (IC_50_ values of these compounds in AGEs were 3.39 and 6.01 *μ*M). These results suggest that the *M. recutita* extract and its constituents may be promising agents for use in the prevention or treatment of diabetic complications.

## 1. Introduction

Persistent hyperglycemia induces abnormal changes, such as increased formation of advanced glycation end products (AGEs) and polyol pathway flux, and the overactivation of protein kinase C isoforms [1]. Diabetic complications including neuropathy, nephropathy, cataracts, and retinopathy are considered to be caused by the accumulation of sorbitol, which is produced from glucose by aldose reductase in the polyol pathway [2]. Aldose reductase (AR, EC 1.1.1.21) catalyzes the reduction of glucose to the corresponding sugar alcohol, sorbitol, which is subsequently metabolized to fructose by sorbitol dehydrogenase [3]. AR is present in almost all mammalian cells, especially in lens, retina and sciatic nerves, which are thus affected by diabetic complications [4]. Increased polyol pathway flux leads to the accumulation of sorbitol in the lens fiber, thus causing an influx of water, generation of osmotic stress, and cataract formation [5].

Reducing sugars can react nonenzymatically with the amino groups of proteins to form reversible Schiff bases. These early glycation products undergo further complex reactions such as rearrangement, dehydration, and condensation to become irreversibly cross-linked, fluorescent derivatives termed AGEs [6]. The formation and accumulation of AGEs in various tissues have been reported to progress at an accelerated rate under hyperglycemic conditions with oxidative stress [7]. This induces oxidative stress and has deleterious effects on various cellular functions Therefore, protein glycation reactions leading to AGEs are thought to be a major cause of different diabetic complications and inhibition of AGE formation could be a novel therapeutic target to prevent complications in diabetes [8].

Flavonoids obtained from natural extracts were reported to have strong AR inhibitory activity and may improve symptoms associated with diabetic complications [9, 10]. In addition, many flavonoid and phenol constituents isolated from natural extracts have various biological activities, including neuroprotective effects in diabetic complications, as well as anti-inflammatory, antidiabetic, and renal protective effects [11–13]. These are distinguished by the number and arrangement of their functional groups and glycosylation. The number of known flavonoids is greater than 4000, and their inhibitory activities are highly varied according to the position and number of functional groups, as well as the glycosylation patterns on flavonoid aglycone [14].

*Matricaria recutita* L. (MR) belongs to the Asteraceae (Compositae) family, one of the largest plant families (23,600 species), and is a herbaceous plant that is indigenous to Europe and Western Asia [15]. MR is a traditional Chinese medicinal herb that has been used in China for centuries to treat various diseases including digestive system illness and diarrhea [16]. The recent work of many research team reported that MR extract also showed antiallergic, anti-inflammatory, and anticancer properties [17]. In addition, MR is a source of phenolic compounds, namely, the flavonoids apigenin, quercetin, kaempferol, and luteolin, as well as their glucosides and also coumarins, which are considered to be the major bioactive compounds of chamomile [18, 19]. Recently studies reported that MR dose-dependently decreased the intestinal absorption of glucose, and *in vitro*, MR treatment showed significant protective effects (liver, kidney, and lipid metabolic parameters) for high fat diet-induced obesity and lipotoxicity in rats [20]. In addition, two new acylated apigenin glucosides that were identified as apigenin-7-*O*-(4^″^-malonyl)-*β*-D-glucopyranoside and apigenin-7-*O*-(4^″^-malonyl-6^″^-acetyl)-*β*-D-glucopyranoside were isolated from edge flowers of *Matricaria chamomilla* [21].

To date, no data have been published on the inhibitory effects of MR extract and its constituents on rat lens AR (RLAR), AGEs, and 2,2-diphenyl-1-picrylhydrazyl (DPPH) radical scavenging regulation. Therefore, the inhibitory activities of compounds isolated from MR on RLAR, AGEs, DPPH, and sorbitol accumulation were investigated to evaluate potential treatments for diabetes-related complications. As well as, we discuss here the structure activity relationships (SAR) of MR extract constituents that could potentially inhibit diabetic complications.

## 2. Materials and Methods

### 2.1. General

^1^H and ^13^C NMR spectra and correlation NMR spectra such as COSY, HMBC, HMQC, and DEPT were obtained from a Bruker Avance DPX 400 (or 600) spectrometer. These spectra were obtained at operating frequencies of 400 MHz (^1^H) and 100 (or 150) MHz (^13^C) with CD_3_OD, and TMS was used as an internal standard. Chemical shifts were reported in *δ* values.

### 2.2. Chemicals and Reagents

L-Ascorbic acid, DPPH, dimethylsulfoxide (DMSO), nicotinamide adenine dinucleotide phosphate (NADPH), DL-glyceraldehyde dimer, bovine serum albumin, methylglyoxal, quercetin, aminoguanidine, sodium phosphate dibasic anhydrous, sodium dihydrogen phosphate, ammonium sulfate, potassium dihydrogen phosphate, sodium hydroxide, sorbitol, and glucose were purchased from Sigma-Aldrich (St. Louis, MO, USA). Sephadex LH-20 was purchased from Wako GE Heakthcare (Milwaukee, WI, USA). All solvents and CD_3_OD used the analytical grade of Sigma-Aldrich (St. Louis, MO, USA).

### 2.3. Plant Materials

Dried MR leaves (Asteraceae) was obtained from local markets in the department of La Libertad in Peru in May 2015. A voucher was deposited at the Center for Efficacy Assessment and Development of Functional Foods and Drugs, Hallym University (P2016-MR). The specimen was authenticated by Paul H. Gonzales Arce in Museo de Historia Natural Universidad Nacional Mayor de San Marcos, Lima, Peru.

### 2.4. Extraction and Isolation

A dried MR leaf (50 g) was extracted with 70% methanol (MeOH, 0.5 L × 2 times) for 3 h at room temperature. The combined filtrates were concentrated to dryness *in vacuo* at 40°C. The extract showed strong inhibitory effects on RLAR, AGEs, and DPPH radical scavenging activity. This extract (3 g) therefore underwent chromatography on a Sephadex LH-20 column using MeOH as the eluent to obtain 16 pooled fractions (MR-SFrac 1–16). Compounds 1 (3.3 mg), 2, and 3 (5.8 mg each) were obtained directly from MR-SFrac 3, 5, and 7, respectively. MR-SFrac 9 was purified to yield the compound 4 (3.3 mg) and 5 (9.3 mg) by recycling HPLC with a gradient system from 20% to 35% MeOH. MR-SFrac 10 and 11 were further fractionated by Sephadex LH-20 with 70% MeOH to obtain compounds 6 (6.1 mg) and 7 (1.3 mg). MR-SFrac 13–15 were further fractionated by Sephadex LH-20 with acetone to obtain compounds 8 (5.5 mg) and 9 (1.6 mg). Compound 10 (1.9 mg) was isolated via silica-gel column chromatography and eluted with a solvent mixture of methyl chloride and MeOH (from 20 : 0 to 1 : 1, *v*/*v*).

### 2.5. Experimental Animals

Experimental animals used in this study were ten male Sprague-Dawley rats with body weight of 250–280 g purchased from Koatech Inc. (Seoul, Korea). They were adapted to a breeding environment of 23 ± 1°C, with 60 ± 5% humidity, below 60 phones, less than 20 ppm odor, 150–300 lux illumination, and 12 hour light and shade cycle for one week with sufficient food and water. Experiments with animals, as well as their breeding and management, were conducted in accordance with the *Guide for the Care and Use of Laboratory Animals*, and experiments were performed with the authorization of the Ethics Committee of Hallym University (Hallym-2016-03). Experiments were performed during the light phase of the cycle (10:00–17:00). The rats were anesthetized prior to the removal of the lenses,and the lenses of both eyes were removed from the rats for AR experiment. The length of time between the removal of lenses and euthanasia is 2–5 min and after the removal of lenses, the animals were immediately euthanized by carbon dioxide (CO_2_) inhalation.

### 2.6. Preparation of RLAR Homogenate

Crude RLAR was prepared as follows: lenses were removed from Sprague-Dawley rats (weighing 250–280 g) and frozen at −70°C until use. Noncataractous transparent lenses were pooled and a homogenate was prepared in 0.1 M phosphate buffered saline (pH 6.2). The RLAR homogenate was then centrifuged at 10,000*g* for 20 min at 4°C in a refrigerated centrifuge. The supernatant was collected and used as RLAR [22].

### 2.7. Determination of RLAR Inhibition

A total of 531 *μ*L of 0.1 M potassium buffer (pH 7.0), 90 *μ*L of NADPH solution (1.6 mM in potassium buffer), 90 *μ*L of RLAR homogenate (6.5 U/mg), 90 *μ*L of ammonium sulfate solution (4 M in potassium buffer), and 90 *μ*L of DL-glyceraldehyde (25 mM in potassium buffer) were mixed with 9 *μ*L of different concentrations of samples (1–0.1 mg/mL in DMSO, less than 1% in total mixture) in a cuvette, and the activity of RLAR was assessed spectrophotometrically by measuring the decrease in NADPH absorbance at 340 nm for 3 minutes using a spectrophotometer (SECOMAM, Ales Cedex, France). Quercetin was used as the positive controls. The inhibition of RLAR (%) was calculated using the following equation: [1 − (△*A* sample/min)–(△*A* blank/min)/(△*A* control/min) − (△*A* blank/min)] × 100%, where △*A* sample/min is the decrease in absorbance over 3 min with reaction solution, test sample, and substrate and △*A* control/min is the same but with DMSO (less than 1% in total mixture) instead of test sample [23].

### 2.8. Methylglyoxal-Bovine Serum Albumin Assay Investigating AGE Formation

Bovine serum albumin (50 mg/mL) was incubated with methylglyoxal (100 mM) in sodium phosphate buffer (0.1 M, pH 7.4) in the presence of various concentrations of the compounds (including a control) at 37°C for 24 h. Then the fluorescent intensity was measured at an excitation wavelength of 355 nm and an emission wavelength of 460 nm with a luminescence spectrometer LS50B (PerkinElmer Ltd., Buckinghamshire, England). The DMSO used as vehicle was found to have no effect on the reaction. All reagents and samples were sterilized by filtration through 0.2 mm membrane filters [23].

### 2.9. Evaluation of DPPH Free Radical Scavenging Capacity

DPPH, a stable free radical, was used to determine the free radical-scavenging activity of the extracts. Briefly, a 0.32 mM DPPH solution in MeOH were prepared, and 180 *μ*L of this solution was mixed with 30 *μ*L of each sample at concentrations of 0.05–1.0 mg/mL in DMSO. After 20 min of incubation in the dark, the decrease in the absorbance of the solution was measured at 570 nm on a microplate reader (EL800 Universal Microplate reader, Bio-Tek instruments, Winooski, VT, USA). DPPH radical-scavenging activity was expressed as the percentage inhibition (%) of DPPH in this assay system and was calculated as (1 − B/A) × 100, where A and B are the activities of DPPH without and with the test material, respectively [24].

### 2.10. Lens Culture and Intracellular Sorbitol Measurement

Lens isolated from 10-week old Sprague-Dawley rats were cultured for 6 d in TC-199 medium containing 15% fetal bovine serum, 100 units/mL penicillin, and 0.1 mg/mL streptomycin, under sterile conditions and an atmosphere of 5% CO_2_ and 95% air at 37°C. Samples were dissolved in DMSO. The lens were divided into three groups (each group *n* = 3) and cultured in medium containing 30 mM glucose and RLAR-active compounds. Each lens was placed in a well containing 2.0 mL medium. Sorbitol was identified by HPLC after its derivatization by reaction with benzoic acid to form a fluorescent compound [25].

### 2.11. Statistical Analysis

Inhibition rates were calculated as percentages (%) with respect to the control value, and the IC_50_ value was defined as the concentration at which 50% inhibition occurred. Data are expressed as mean values ± standard deviation of triplicate experiments. Data were analyzed using SPSS version 19.0 software. The comparison of mean values was carried out by Student's unpaired *t*-test or one-way analysis of variance (ANOVA), as appropriate; *p* < 0.05 was considered statistically significant.

## 3. Results

### 3.1. Structure Analysis of Isolated Compounds

**Figure 1:**
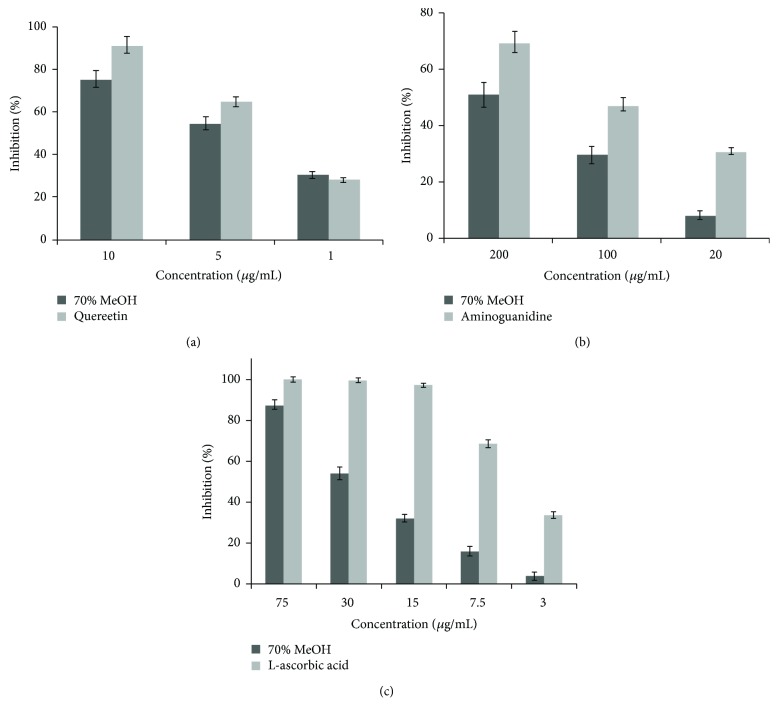
The inhibitory activities by MR crude extract on rat lens aldose reductase (a), advanced glycation end products (b), and DPPH radical-scavenging (c) in various concentrations.

**Figure 2:**
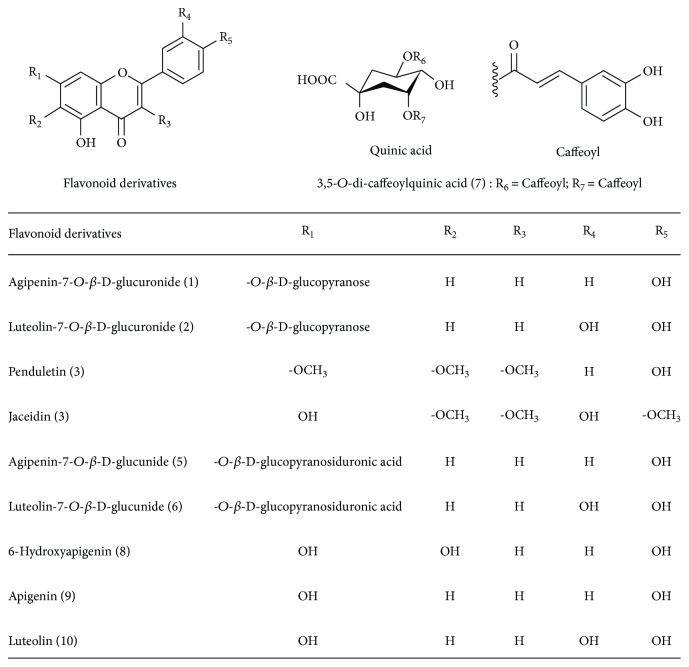
The chemical structures of compounds isolated from *Matricaria recutita* L.

The MR extract was found to exhibit strong RLAR, AGEs, and antioxidant inhibitory activities, with an IC_50_ of 4.61, 189.08, and 32.39 *μ*g/mL (Figure 1 and Table 1), respectively. Since this result suggests the likely presence of many AR inhibitors (ARIs) in the extract, attention should be focused on isolating these from this fraction. In order to identify the active compounds from MR, the extract was dissolved in methanol and subjected to repeated chromatography on Sephadex LH-20, silica gel, and reverse-phase C18 columns, to yield compounds 1–10. The structures of isolated compounds were elucidated based on 1-dimensional (^1^H and ^13^C NMR) and 2-dimensional NMR (HMQC and HMBC) spectral data, by comparing with published spectral data, electronic impact (EI), and fast atom bombardment (FAB) mass spectrometry (MS) data. Isolated compounds were identified as apigenin-7-*O*-*β*-D-glucoside (1), luteolin-7-*O*-*β*-D-glucoside (2), penduletin (3), jaceidin (4), apigenin-7-*O*-*β*-D-glucuronide (5), luteolin-7-*O*-*β*-D-glucuronide (6), 3,5-*O*-di-caffeoylquinic acid (7), 6-hydroxyapigenin (8), apigenin (9), and luteolin (10). The chemical structures of compounds 1–10 isolated from MR are shown in Figure 2 [14–29].

Compound (1). FAB-MS *m/z* 433 [M + H]^+^. ^1^H-NMR (400 MHz, CD_3_OD, *δ*_H_) *δ* 7.87 (2H, d, *J* = 8.41 Hz, H-2′/6′), 6.91 (2H, d, *J* = 8.41 Hz, H-3′/5′), 6.87 (1H, s, H-3), 6.81 (1H, d, *J* = 1.73 Hz, H-8), 6.44 (1H, d, *J* = 1.73 Hz, H-6), 5.15 (1H, d, *J* = 7.51 Hz, H-1^″^), 3.97–3.13 (6H, m, H-2^″^, 3^″^, 4^″^, 5^″^ and 6ab^″^), ^13^C-NMR (100 MHz, CD_3_OD, *δ*_c_) *δ* 180.1 (C-4), 166.1 (C-7), 162.4 (C-2), 161.8 (C-5), 160.1 (C-4′), 157.1 (C-9), 125.9 (C-2′/6′), 121.7 (C-1′), 116.3 (C-3′/5′), 104.8 (C-10), 101.5 (C-3), 98.6 (C-1^″^), 97.9 (C-6), 94.3 (C-8), 77.4 (C-3^″^), 76.8 (C-5^″^), 74.0 (C-2^″^), 69.8 (C-4^″^), 63.8 (C-6^″^).

Compound (2). FAB-MS *m/z* 449 [M + H]^+^. ^1^H-NMR (400 MHz, CD_3_OD, *δ*_H_) 7.51 (1H, dd, *J* = 8.13, 2.07 Hz, H-6′), 7.44 (1H, d, *J* = 2.04 Hz, H-2′), 6.88 (1H, d, *J* = 8.12 Hz, H-5′), 6.84(1H, s, H-3), 6.73 (1H, d, *J* = 2.10 Hz, H-8), 6.49 (1H, d, *J* = 2.10 Hz, H-6), 5.11 (1H, d, *J* = 7.39 Hz, H-1^″^), 3.85–3.36 (6H, m, H-2^″^, 3 ^″^, 4^″^, 5^″^ and 6ab^″^), ^13^C-NMR (100 MHz, CD_3_OD, *δ*_c_) *δ* 181.2 (C-4), 164.6 (C-7), 163.8 (C-2), 161.3 (C-5), 158.2 (C-9), 153.4 (C-4′), 147.8 (C-3′), 125.1 (C-6′), 122.7 (C-1′), 117.0 (C-5′), 115.7 (C-2′), 106.5 (C-10), 103.2 (C-3), 101.5 (C-1^″^), 99.7 (C-6), 96.7 (C-8), 77.1 (C-3^″^), 76.1 (C-5^″^), 74.3 (C-2^″^), 71.1 (C-4^″^), 63.3 (C-6^″^).

Compound (3). FAB-MS *m/z* 345 [M + H]^+^. ^1^H-NMR (400 MHz, CD_3_OD, *δ*_H_) *δ* 7.73 (2H, d, *J* = 8.31 Hz, H-2′/6′), 6.74 (2H, d, *J* = 8.13 Hz, H-3′/5′), 6.81 (1H, s, H-8), 3.81 (9H, s, -OCH_3_, H-3/6/7), ^13^C-NMR (100 MHz, CD_3_OD, *δ*_c_) *δ* 177.9 (C-4), 159.4 (C-7), 157.3 (C-4′), 156.1 (C-2), 155.7 (C-9), 153.2 (C-5), 138.3 (C-3), 136.9 (C-6), 129.9 (C-2′/6′), 121.7 (C-1′), 116.7 (C-3′/5′), 104.9 (C-10), 96.2 (C-8), 60.1 (C-6, -OCH_3_), 58.7 (C-3, -OCH_3_), 56.3 (C-7, -OCH_3_).

Compound (4). FAB-MS *m/z* 361 [M + H]^+^. ^1^H-NMR (400 MHz, CD_3_OD, *δ*_H_) *δ* 7.57 (1H, dd, *J* = 8.09, 1.97 Hz, H-6′), 6.83 (1H, d, *J* = 8.07 Hz, H-5′), 6.77 (1H, d, *J* = 2.00 Hz, H-2′), 6.73 (1H, s, H-8), 3.80 (9H, s, −OCH_3_, H-3/4′/6), ^13^C-NMR (100 MHz, CD_3_OD, *δ*_c_) *δ* 178.1 (C-4), 157.8 (C-7), 155.3 (C-9), 154.6 (C-2), 151.3 (C-5), 148.9 (C-4′), 147.3 (C-3′), 138.5 (C-3), 130.9 (C-6), 121.7 (C-1′), 120.0 (C-6′), 114.7 (C-2′), 111.1 (C-5′), 104.1 (C-10), 95.7 (C-8), 60.6 (C-6, -OCH_3_), 58.6 (C-3, -OCH_3_), 56.9 (C-4′, -OCH_3_).

Compound (5). FAB-MS *m/z* 447 [M + H]^+^. ^1^H-NMR (400 MHz, CD_3_OD, *δ*_H_) 7.84 (2H, d, *J* = 8.77 Hz, H-2′/6′), 6.98 (2H, d, *J* = 8.73 Hz, H-3′/5′), 6.84 (1H, s, H-3), 6.76 (1H, d, *J* = 1.82 Hz, H-8), 6.49 (1H, d, *J* = 1.82 Hz, H-6), 5.07 (1H, d, *J* = 7.07 Hz, H-1^″^), 3.96 (1H, d, *J* = 9.59 Hz, H-5^″^), 3.60–3.26 (3H, m, H-2^″^, 3^″^ and 4^″^), ^13^C-NMR (100 MHz, CD_3_OD, *δ*_c_) *δ* 180.1 (C-4), 171.5 (C-6^″^), 166.6 (C-7), 163.8 (C-2), 161.7 (C-5), 158.5 (C-4′), 155.7 (C-9), 128.9 (C-2′/6′), 122.1 (C-1′), 118.0 (C-3′/5′), 106.5 (C-10), 102.8 (C-3), 101.37 (C-1^″^), 99.2 (C-6), 96.3 (C-8), 78.0 (C-3^″^), 75.1 (C-5^″^), 73.9 (C-2^″^), 71.8 (C-4^″^).

Compound (6). FAB-MS *m/z* 463 [M + H]^+^. ^1^H-NMR (400 MHz, CD_3_OD, *δ*_H_) *δ* 7.55 (1H, dd, *J* = 8.10, 2.08 Hz, H-6′), 7.42 (1H, d, *J* = 2.07 Hz, H-2′), 6.94 (1H, d, *J* = 8.10 Hz, H-5′), 6.82 (1H, s, H-3), 6.79 (1H, *J* = 2.30 Hz, H-8), 6.56 (1H, d, *J* = 2.30 Hz, H-6), 5.11 (1H, d, *J* = 7.27 Hz, H-1^″^), 4.08 (1H, d, *J* = 9.50 Hz, H-5^″^), 3.51–3.27 (3H, m, H-2^″^, 3^″^ and 4^″^), ^13^C-NMR (100 MHz, CD_3_OD, *δ*_c_) *δ* 180.1 (C-4), 172.1 (C-6^″^), 165.8 (C-7), 162.8 (C-2), 160.1 (C-5), 152.7 (C-9), 150.7 (C-4′), 146.7 (C-3′), 117.0 (C-6′), 116.82 (C-1′), 114.1 (C-5′), 112.1 (C-2′), 102.9 (C-10), 100.9 (C-3), 101.1 (C-1^″^), 98.8 (C-6), 95.1 (C-8), 76.8 (C-3^″^), 73.4 (C-5^″^), 74.3 (C-2^″^), 72.5 (C-4^″^).

Compound (7). FAB-MS *m/z* 517 [M + H]^+^. ^1^H-NMR (400 MHz, CD_3_OD, *δ*_H_) *δ* 7.63, 7.61 (1H each, d, *J* = 16.01 Hz, H-7/H-7′), 7.12 (2H, brs, H-2/H-2′), 6.92 (2H, dd, *J* = 8.14, 2.01 Hz, H-6/H-6′), 6.80 (2H, dd, *J* = 7.80, 1.22 Hz, H-5/H-5′), 6.37, 6.29 (1H each, d, *J* = 16.01 Hz, H-8/H-8′), 5.55–5.39 (2H, m, H-3/H-5), 4.01 (1H, dd, *J* = 9.71, 3.26 Hz, H-4), 2.39–2.17 (4H, m, H-2/H-6). ^13^C-NMR (100 MHz, CD_3_OD, *δ*_c_) *δ* 176.7 (COOH), 167.9, 167.5 (C-9/C-9′), 148.4, 148.2 (C-4/C-4′), 146.8, 146.6 (C-7/C-7′), 145.8 (C-3/C-3′), 126.9, 126.7 (C-1/C-1′), 121.9, 121.5 (C-6/C-6′), 117.8 (C-5/C-5′), 115.7, 115.6 (C-8/C-8′), 115.0 (C-2/C-2′), 73.4 (C-1), 71.9 (C-3), 70.9 (C-5), 69.8 (C-4), 37.9 (C-6), 36.7 (C-2).

Compound (8). FAB-MS *m/z* 287 [M + H]^+^. ^1^H-NMR (400 MHz, CD_3_OD, *δ*_H_) *δ* 7.74 (2H, d, *J* = 8.21 Hz, H-2′/H-6′), 6.84 (2H, d, *J* = 8.14 Hz, H-3′/H-5′), 6.76 (1H, s, H-3), 6.40 (1H, s, H-8); ^13^C-NMR (100 MHz, CD_3_OD, *δ*_c_) *δ* 181.6 (C-4), 164.3 (C-2), 159.7 (C-7), 158.6 (C-4′), 157.3 (C-5), 153.1 (C-9), 145.9 (C-6), 129.0 (C-2′/C-6′), 120.4 (C-1′), 115.6 (C-3′/C-5′), 106.1 (C-10), 102.3 (C-3), 94.6 (C-8).

Compound (9). EI-MS *m/z* 271 [M + H]^+^. ^1^H-NMR (400 MHz, CD_3_OD, *δ*_H_) *δ* 7.82 (2H, d, *J* = 8.15 Hz, H-2′/H-6′), 6.97 (2H, d, *J* = 8.15 Hz, H-3′/H-5′), 6.79 (1H, s, H-3), 6.58 (1H, d, *J* = 2.11 Hz, H-8), 6.39 (1H, *J* = 2.11 Hz, H-6); ^13^C-NMR (100 MHz, CD_3_OD, *δ*_c_) *δ* 180.0 (C-4), 165.7 (C-2), 163.9 (C-7), 160.9 (C-5), 160.2 (C-4′), 158.6 (C-9), 129.1 (C-2′/C-6′), 120.9 (C-1′), 115.7 (C-3′/C-5′), 101.3 (C-10), 100.1 (C-3), 98.8 (C-6), 94.0 (C-8).

Compound (10). EI-MS *m/z* 287 [M + H]^+^. ^1^H-NMR (400 MHz, CD_3_OD, *δ*_H_) *δ* 7.51 (1H, dd, *J* = 9.17, 2.02 Hz, H-6′), 7.31(1H, d, *J* = 2.02 Hz, H-2′), 6.81 (1H, d, *J* = 9.21 Hz, H-5′), 6.68 (1H, s, H-3), 6.47 (1H, d, *J* = 2.03 Hz, H-8), 6.18 (1H, d, *J* = 2.03 Hz, H-6). ^13^C-NMR (100 MHz, CD_3_OD, *δ*_c_) *δ* 180.6 (C-4), 163.7 (C-2), 162.1 (C-7), 160.7 (C-5), 158.5 (C-9), 148.2 (C-4′), 144.9 (C-3′), 120.5 (C-6′), 118.7 (C-1′), 116.2 (C-5′), 113.8 (C-2′), 104.2 (C-10), 102.7 (C-3), 99.2 (C-6), 96.8 (C-8).

### 3.2. AR Inhibitory Activities of the Isolated Compounds

The inhibitory activities of compounds 1–10 on RLAR were evaluated. As shown in Table 2, compounds 6 and 7 showed the strongest inhibition against RLAR (IC_50_ = 0.85 and 0.72 *μ*M, resp.). In addition, compounds 2 (IC_50_ = 1.12 *μ*M), 5 (IC_50_ = 1.16 *μ*M), 9 (IC_50_ = 1.71 *μ*M), and 10 (IC_50_ = 1.42 *μ*M) were found to possess significant RLAR inhibitory activity *in vitro* (2 > 5 > 10> 9), compared to quercetin (IC_50_ = 1.21 *μ*M), a well-known ARI. Compounds 3, 4, and 8 were inactive.

### 3.3. Inhibitory Activities of AGEs

The methylglyoxal-BSA assay was used specifically to investigate inhibitors of protein glycation formation in MR extract and was performed according to the method characterized by Li et al. [30]. The MR extract showed high AGE inhibitory activity with an IC_50_ value of 189.08 *μ*g/mL. In addition, we compared inhibition of the formation of advanced glycation by compounds 1–10 with that achieved by AG, a well-known AGE inhibitor. As shown in Table 2, compounds 6 and 10 (luteolin-7-*O*-*β*-D-glucuronide and luteolin) had IC_50_ values of 3.39 and 6.01 *μ*M, respectively, and were found to be more effective than AG in inhibiting the formation of advanced glycation, while other compounds were inactive and showed varied low inhibitory effects ranging from 9.06–16.25% at a concentration of 20 *μ*g/mL. Our results showed that compounds 6 and 10, which contain a glucuronide at position 7 in the A ring and di-hydroxyl groups in the B ring of the flavonol skeleton, exhibited the highest AGE inhibitory activity.

### 3.4. Antioxidant Activities of the Isolated Compounds

The MR extract exhibited potent inhibition on DPPH free radical-scavenging activity (32.39 *μ*g/mL) compared to the positive control L-ascorbic, which had an IC_50_ value of 6.60 *μ*g/mL. The scavenging activities of the ten compounds isolated from MR were evaluated using the same method (Table 2). Of the tested compounds, compounds 2 and 6 had the highest IC_50_ values: 7.24 and 8.92 *μ*M, respectively. Compounds 5, 7, 9, and 10 also showed strong scavenging activity with IC_50_ values of 10.58–15.63 *μ*M, compared to the positive control, L-ascorbic acid (IC_50_ = 3.75 *μ*M). Compounds 3, 4, and 8 had almost no effect on DPPH free radical scavenging activity.

### 3.5. Inhibitory Activities of Active Compounds on Sorbitol Accumulation

We also investigated the effects of RLAR inhibitory compounds on sorbitol accumulation in isolated rat lens (results shown in Table 3). Compounds 1, 2, 5, 6, 7, 9, and 10 effectively inhibited sorbitol accumulation by 51.02, 95.23, 80.27, 91.83, 86.39, 87.07, and 91.83% at concentration of 5 *μ*g/mL, respectively. The positive control (quercetin) inhibited sorbitol accumulation in rat lens by 85.71% and reduced sorbitol levels in culture medium containing a high glucose concentration.

### 3.6. Interaction Analysis of Active Compounds Isolated with AR

**Figure 3:**
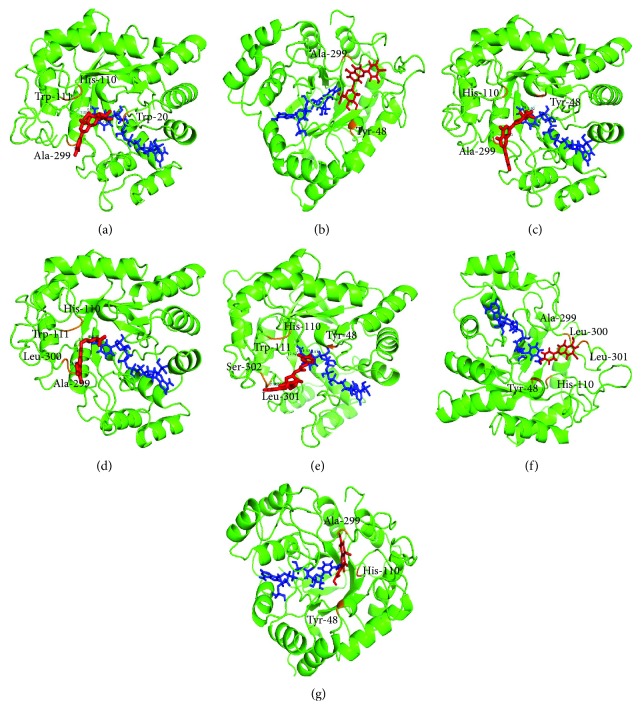
Docking models of apigenin-7-*O*-*β*-D-glucoside (a), luteolin-7-*O*-*β*-D-glucoside (b), apigenin-7-*O*-*β*-D-glucuronide (c), luteolin-7-*O*-*β*-D-glucuronide (d), 3,5-*O*-di-caffeoylquinic acid (e), apigenin (f), and luteolin (g). The structure of aldose reductase is in green; the structures of the ligands are in red; the interactions of the residues with the ligands are shown in orange.

To explore the binding of flavonoids and AR, molecular docking studies were carried. Docking interactions showed that the flavonoids isolated from MR bind stably with AR (Figure 3). Compounds bind to the active site of AR at Ser-302; His-110; Ala-299; Leu-301 and 302, and Trp-20, 48, and 111 residues. All seven compounds occupied the active site and interacted with the surrounding residues at different orientations. The molecular docking method can reveal the nature of ligand binding at active site for various compounds. Our molecular docking simulation suggested that the strategy for screening AR inhibitor from natural products is reliable and can be used to distinguish the specific inhibitors from false positives.

## 4. Discussion

The flavonoids and derivatives are an interesting group of natural products that are found in various widely distributed plants, and most of these compounds are isolated from medicinal plants [31, 32]. Previous investigations into the inhibitory activities of flavonoids and their derivatives reported that luteolin (10) and luteolin-5-*O*-*β*-D-glucopyranoside isolated from *Cirsium maackii* (A perennial thistle of Asteraceae family) showed inhibitory effects on RLAR comparable to those of the positive control (quercetin). This study indicated that the inhibitory activity of luteolin-5-*O*-*β*-D-glucopyranoside on RLAR was almost 1.58 times greater than that of its precursor [33]. In our results, luteolin-7-*O*-*β*-D-glucoside (2) (monoglycosylation) showed to be higher than luteolin (10) (aglycone) on RLAR. Monoglycosylation and diglycosylation at luteolin (10) elevated its inhibitory potency significantly, suggesting that the RLAR inhibitory activity of luteolin (10) is strongly related to the number and position of sugar moieties. Luteolin-7-*O*-*β*-D-glucoside (2) and luteolin-7-*O*-rutinoside were isolated from *Colocasia esculenta* by Li et al. [34], and a number of sugar moieties at the same positions in the flavonoid skeleton were shown to have different inhibitory activities on RLAR [34]. However, the effects of these products on sorbitol accumulation were not reported. Jung et al. (2011) suggested that the addition of a sugar group to the flavonoid skeleton in position 3 (quercetin-3-*O*-glucoside and quercetin-3-*O*-*β*-D-galactoside) may be responsible for a loss of RLAR inhibitory activity compared to its precursor (quercetin) [35]. In addition, quercetin derivatives (quercetin-3-*O*-D-glucoside, quercetin-3-*O*-*β*-D-glucuronide, quercetin-3-*O*-*β*-D-galactoside, and quercetin-3-*O*-*β*-D-rutinoside) isolated from the extracts of *Nelumbo nucifera* leaves exhibited the most potent inhibitory activity in RLAR, AGEs, and oxidative stress [35]. This work indicates that sugar moieties in flavonoid skeletons may be implicated in the potency of RLAR inhibitory effects. The RLAR inhibitory activities of flavonoid compounds 1, 2, 6, 7, 9, and 10 isolated from plant sources were as follows: compound 1 (23.0 *μ*M), compound 2 (0.99 *μ*M), compound 6 (3.1 *μ*M), compound 7 (0.19 *μ*M), compound 9 (2.2 *μ*M), and compound 10 (0.45 *μ*M) [36–39]. These reported data were similar to the SAR data of our flavonoids on RLAR.

Compounds 1 and 2 are glucosides, 3 and 4 are methoxy aglycones, compounds 5 and 6 are glucuronides, and compounds 8–10 are aglycones. Flavonoid glucuronides showed higher inhibitory activities against AR than their precursor and glycosides. Furthermore, di-hydroxy B-ring flavonoids (compounds 2, 6, and 10) showed higher inhibitory activities against AR than mono-hydroxy B-ring flavonoids (compounds 1, 5, and 9). On the other hand, methoxy (compounds 3 and 4) and tri-hydroxy A-ring flavonoids (compound 8) did not show inhibitory activities against RLAR. In addition, Matsuda et al. [39] reported that the RLAR inhibitory effects of flavonoid derivatives depend on the number and site of hydroxyl, methoxyl, and sugar moieties in the aromatic ring of the flavonoid skeleton [39]. On the other hand, other compounds (excluding 3, 4, and 8) showed similar activities on DPPH radical-scavenging activities. Based on these results, a significant relationship between RLAR inhibitory activities and protective properties against oxidative stress was observed in flavonoids.

According to the many structural properties of flavonoids, the inhibition of AGE formation has been reported [40]. Compound 6 showed stronger antiglycation effect than both compounds 10 (luteolin) and 2. Increasing the number of glucuronides at position 7 of the A ring (compound 6) in the skeleton of compound 10 increased its inhibitory activities on protein glycation. Jung et al. [41] previously reported that the number of hydroxyl groups at positions 3 and 4 of the B ring in compound 10 increased its inhibitory activities against each stage of protein glycation. Compounds 3, 4, and 8 did not show inhibitory activities on the formation of advanced glycation. For this reason, our SAR data suggest that a hydroxyl group at position 3 of the B ring and a glucuronide group at position 7 of the A ring may contribute to the AGE inhibitory activity. The flavonoids isolated from MR do have an established SAR to explain the antiglycation activity demonstrated in the assays above. Based on these results, flavonoids showed a significant relationship between AGE inhibitory activities and functional groups in the flavonoid skeleton. Compounds 1, 2, 5, 6, 7, 9, and 10 showed different RLAR inhibitory activities (compound 7 > 6 > 2 > 5 > 10> 9 > 1). On the other hand, the strong inhibition of sorbitol accumulation was observed, in the following order: compound 2 (95.23%) > 6 and 10 (91.83%) > 9 (87.07%) > 7 (86.39%) > 5 (80.27%) > 1 (51.02%). Previously, Kim et al. [42], Lee et al. [43], and Kim et al. [44] reported that the flavonoid derivatives isolated from extracts of *Paulownia coreana*, *Quercus acutissima*, and *Chamaecyparis obtusa* exerted inhibitory effects on sorbitol accumulation based on the RLAR assay.

In this study, different inhibitory activities were seen *in vitro* and ex vivo and were related to the structures of the flavonoids. Therefore, this result suggests that AR inhibition and sorbitol accumulation may be affected by the structures of the flavonoids. However, the mechanism of MR and its constituents on inhibitory effects of AR and AGEs formation have not yet been found. Therefore, more physiological studies of the MR will be needed for the development of phytomedicine and functional food source.

**Figure 4:**
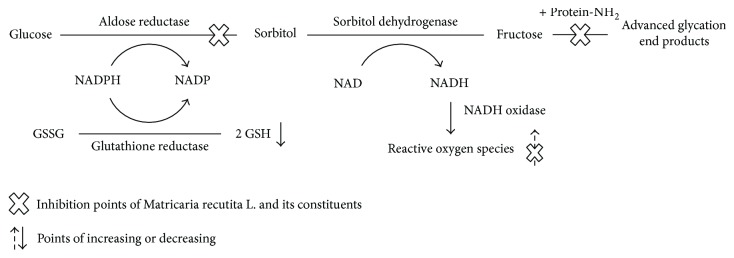
Inhibition points of *Matricaria recutita* L. and its constituents on polyol pathway. GSH: glutathione; GSSG: glutathione disulfide; NAD: nicotinamide adenine dinucleotide; NADH: oxidoreductase-induced nicotinamide adenine dinucleotide; NADP: nicotinamide adenine dinucleotide phosphate; NADPH: oxidoreductase-induced nicotinamide adenine dinucleotide phosphate.

In the present study, the RLAR, AGE, and DPPH radical-scavenging inhibitory activities of MR and its constituents were investigated. MR and its constituents showed high inhibitory activities in three *in vitro* assays (Figure 4), and their considerable beneficial effects on diabetic complications would make MR a good ingredient in functional foods. Furthermore, the flavonoids (compounds 1, 2, 5, 6, 9, and 10) and polyphenol (compound 7) isolated from MR showed potent inhibitory activities on sorbitol accumulation in isolated rat lens. This research may provide fundamental knowledge for the development of RLAR, AGE, and antioxidant inhibitors from MR and/or its components. These results suggest that MR and its constituents can be potent functional food ingredients as RLAR and AGE inhibitors and can be used as naturotherapy for diabetic complications, including oxidative stress.

## Figures and Tables

**Table 1:** The inhibitory activities of MR crude extract on rat lens aldose reductase (RLAR), DPPH radical scavenging, and advanced glycation end products (AGEs).

Entry	IC_50_ (*μ*g/mL)^1)^		
	RLAR	DPPH	AGEs
70% MeOH	4.61 ± 0.29^b^	32.39 ± 1.28^b^	189.08 ± 4.19^b^
Quercetin^2)^	3.65 ± 0.10^a^	—	—
L-Ascorbic acid^3)^	—	6.60 ± 0.33^a^	—
Aminoguanidine^4)^	—	—	109.10 ± 3.47^a^

^1)^The IC_50_ values are defined as mean ± relative standard derivation (RSD) of half-maximal inhibitory concentrations obtained from three independent experiments performed in duplicate, and the range of the inhibitor concentrations adopted to evaluate IC_50_ was prepared as follows: (1) RLAR: 1, 5, and 10 *μ*g/mL; (2) DPPH: 15, 30, and 75 *μ*g/mL; and (3) AGEs: 20, 100, and 200 *μ*g/mL. ^2)–4)^Quercetin, L-ascorbic acid, and aminoguanidine are the positive control for RLAR inhibition, DPPH scavenging, and AGEs inhibition. Values within a column marked with different letters are significantly different from each other (*p* < 0.05).

**Table 2:** Inhibitory activities of compounds isolated from *Matricaria recutita* L. on rat lens aldose reductase (RLAR), DPPH radical scavenging, and advanced glycation end products (AGEs).

Compounds	IC_50_ (*μ*M)^1)^		
	RLAR	DPPH	AGEs
Agipenin-7-*O*-*β*-D-glucoside (1)	4.25 ± 0.07^d^	>25.0	NI
Luteolin-7-*O*-*β*-D-glucoside (2)	1.12 ± 0.02^b^	7.24 ± 0.38^b^	NI
Penduletin (3)	NI^2)^	NI	NI
Jaceidin (4)	NI	NI	NI
Agipenin-7-*O*-*β*-D-glucuronide (5)	1.16 ± 0.04^b^	10.58 ± 0.47^bc^	NI
Luteolin-7-*O*-*β*-D-glucuronide (6)	0.85 ± 0.02^a^	8.92 ± 0.21^b^	3.39 ± 0.17^a^
3,5-*O*-di-caffeoylquinic acid (7)	0.72 ± 0.02^a^	12.34 ± 0.63^bc^	NI
6-Hydroxyapigenin (8)	NI	NI	NI
Apigenin (9)	1.72 ± 0.04^c^	15.63 ± 0.34^c^	NI
Luteolin (10)	1.42 ± 0.03^bc^	11.53 ± 0.38^bc^	6.01 ± 0.38^b^
Quercetin^3)^	1.21 ± 0.04^b^	—	—
L-Ascorbic acid^4)^	—	3.75 ± 0.17^a^	—
Aminoguanidine^5)^	—	—	98.69 ± 5.31^c^

^1)^The IC_50_ values are defined as mean ± relative standard derivation (RSD) of half-maximal inhibitory concentrations obtained from three independent experiments performed in duplicate and the range of the inhibitor concentrations adopted to evaluate IC_50_ was prepared as follows: (1) RLAR: 1, 5, and 10 *μ*g/mL; (2) DPPH: 15, 30, and 75 *μ*g/mL; and (3) AGEs: 10, 25, and 50 *μ*g/mL. ^2)^NI: no inhibition. ^3)–5)^Quercetin, L-ascorbic acid, and aminoguanidine are the positive control for RLAR inhibition, DPPH scavenging, and AGEs inhibition. Values within a column marked with different letters are significantly different from each other (*p* < 0.05).

**Table 3:** Inhibitory effects of rat lens aldose reductase-active compounds of *Matricaria recutita* L. on sorbitol accumulation in rat lens.

Compounds	Sorbitol content (mg)/lens wet weight (g)	Inhibition (%)
Sorbitol free by G free	No detection	—
Control by G	1.47 ± 0.02	—
Quercetin^1)^ by G + quercetin	0.21 ± 0.01^a^	85.71 ± 3.23^a^
Agipenin-7-*O*-*β*-D-glucoside (1)	0.72 ± 0.01^c^	51.02 ± 1.68^c^
Luteolin-7-*O*-*β*-D-glucoside (2)	0.07 ± 0.01^a^	95.23 ± 8.18^a^
Agipenin-7-*O*-*β*-D-glucuronide (5)	0.29 ± 0.01^b^	80.27 ± 2.78^b^
Luteolin-7-*O*-*β*-D-glucuronide (6)	0.12 ± 0.01^a^	91.83 ± 6.74^a^
3,5-*O*-di-caffeoylquinic acid (7)	0.20 ± 0.01^a^	86.39 ± 4.28^a^
Apigenin (9)	0.19 ± 0.01^a^	87.07 ± 4.48^a^
Luteolin (10)	0.12 ± 0.01^a^	91.83 ± 6.87^a^

^1)^Quercetin was used as the positive control. Results are presented as mean ± SD (*n* = 3). Values within a column marked with different letters are significantly different from each other (*p* < 0.05). Samples concentration was used at 5 *μ*g/mL on sorbitol accumulation in rat lens.
